# Oral Potentially Malignant Disorders: An Overview of More than 20 Entities

**DOI:** 10.5681/joddd.2014.002

**Published:** 2014-03-05

**Authors:** Hamed Mortazavi, Maryam Baharvand, Masoumeh Mehdipour

**Affiliations:** ^1^1Associate Professor, Department of Oral and Maxillofacial Medicine, Faculty of Dentistry, Shahid Beheshti University of Medical Sciences, Tehran, Iran

**Keywords:** Precancerous conditions, oral cavity neoplasms, malignancy, leukoplakia, oral cancer

## Abstract

Cancer of the oral cavity accounts for approximately 3% of all malignancies diagnosed annually in 270,000 patients world-wide. Oral cancer is the 12th most common cancer in women and the 6th in men. Many oral squamous cell carcinomas develop from potentially malignant disorders (PMDs). Lack of awareness about the signs and symptoms of oralPMDs in the general population and even healthcare providers is believed to be responsible for the diagnostic delay of these entities. The aim of this article is to update and improve the knowledge of healthcare providers about oral PMDs.

## Introduction

Cancer is the second most common cause of death after heart diseases in developed countries, and the third leading cause of mortality following heart and diarrheal diseases in developing countries.^1^ Cancer of the oral cavity accounts for approximately 3% of all malignancies and found in 270,000 patients annually worldwide.^2,3^ It is the 12th most common cancer in women and the 6th in men.^4^ Almost 4–8.1% of females and 8–8.5% of males may develop oral cancer in their lives.^1,5^ Black males have a higher incidence rate of oral cancer than whites (20.7 cases versus 15.3 per 100,000 annually). In recent years, the incidence rate of oral cancer has increased from 16.8 to 20.7 per 100,000 in blacks, whereas in whites it has declined from 17.5–15.3 persons per year.^6,7^ Oral squamous cell carcinoma (OSCC) comprises 92–95% of alloral cancers.^1^ Although 95% of oral cancers occur in individuals older than 40,^8^ there has been an increase in the incidence of oral cancer in people younger than 40 years from 3% in 1973 to 6% in 1993.^9^ This finding may be related to popularity of high-risk habits such as tobacco and alcohol use in young individuals. Furthermore, the traditional male predominance is less overt in young people with OSSC.^9^ Unfortunately, the five-year survival rate of OSCC has not significantly improved over the past decades and is still about 53–56%.^2^ Many factors are known to have etiologic roles in OSCC such as tobacco smoke, alcohol, phenol, viral, bacterial and fungal infections, electro-galvanic reaction, radiation, genetics, immunosuppression, expression of oncogens, deactivation of tumor suppressor genes and malnutrition.^1,4,5,9-13^

It is noteworthy that many oral squamous cell carcinomas develop from potentially malignant disorders (PMDs).^1,3,14^ Correct diagnosis and timely treatment of PMDs may help prevent malignant transformation in oral lesions.^1^ Lack of awareness about signs and symptoms of oral PMDs among general population and even physicians are believed to be responsible for the diagnostic delay of these entities.^1,3^ The aim of this article is to update and improve the knowledge of healthcare providers about oral PMDs.

###  What are PMDs?

The clinical concept of malignant transformation in oral mucosa has been proposed for more than 100 years. Sir James Paget^15^first described malignant transformation of an oral lesion into tongue carcinomain 1870. Schwimmer^16^also reported the same findingin 1877. Several years later, the term "potentially malignant disorders" was defined by World Health Organization (WHO) as the risk of malignancy being present in a lesion or condition either during the time of initial diagnosis or at a future date. WHO also classified PMDs into two subgroups as follows: a) precancerous lesion, a benign lesion with morphologically altered tissue, which has a greater than normal risk of transforming into malignancy; b) precancerous condition, a disease or patients’ habit that does not necessarily alter the clinical appearance of local tissues but is associated with a greater than normal risk of precancerous lesion or cancer development in that tissue (Table 1).^3,4^ Although little information is available regarding the real prevalence of PMDs in the general population, a commonly accepted prevalence of 1–5% has been reported.^3^ Average age of patients with PMDs is 50–69 years, which is 5 years before occurrence of oral cancer. Unfortunately, in recent years 5% of PMDs has been observed in persons under 30.^1^ Premalignant disorders are usually found on the buccal mucosa, followed by gingivae, tongue and floor of the mouth.^1,16^

**Table 1:** Potentially Malignant Disorders (PMDs)

Premalignant lesions	Premalignant conditions
Leukoplakia	Lichen planus
Erythroplakia	Discoid lupus erythematosus
Proliferative verrucous leukoplakia(PVL)	Epidermolysis bullosa
Viadent leukoplakia	Verruciform xanthoma
Candida leukoplakia	Graft-versus-host-disease
Reverse smokings’ palate	Cheilitis glandularis
Verrucous hyperplasia	Xeroderma pigmentosum
Oral verrucous carcinoma	Syphilis (third stage)
Dyskeratosis congenita	Plummer-Vinson syndrome
Actinic cheilosis	Malnutrition
Keratoacanthoma	Vitamin A, B, C deficiency
Oral submucous fibrosis	Immunosuppressive diseases [AIDS]

Characteristics of precancerous disorders are summarized in Table 2.

**Table 2:** Characteristics of potentially malignant disorders

Disorders	Clinical features	Locations	Risk of malignancy
Leukoplakia	White plaque	Cheeks, lips, gingivae	15.6–39.2%
Early (thin)			NA^*^
Homogenous			1–7%
Verruciform			4–15%
Speckled			18–47%
Erythroplakia	A predominantly red lesion	Mouth floor, tongue, retromolar pad, soft palate	51%
Proliferative verrucous leukoplakia (PVL)	Multifocal white patch or plaque + rough surface projections	Gingivae	63.3–100%
Viadent leukoplakia	White patch or plaque	Gingivae, buccal and labial vestibule	NA
Candida leukoplakia	Firm, white leathery plaques	Cheeks, lips, palate	4–5 times more common than leukoplakia
Smokeless tobacco keratosis	White plaque	Buccal or labial vestibule	NA
Palatal keratosis associated with reverse smoking	White patches and plaques	Palate, tongue	83.3% dysplasia 12.5% SCC
Verrucous hyperplasia	Extensive thick white plaque	Buccal mucosa	68% dysplasia
Oral verrucous carcinoma	Extensive thick white plaque	Buccal mucosa	20%
Dyskeratosis congenita	Oral leukoplakia	Buccal mucosa, tongue, oropharynx	NA
Actinic cheilosis	Diffuse, poorly defined atrophic, erosive, ulcerative or keratotic plaques	Lower lip	6–10%
Keratoacanthoma	Firm,sessile non tender nodule + a central plug of keratin	Lips, tongue, sublingual region	24%
Oral submucous fibrosis	Mucosal rigidity	Buccal mucosa, retromolar area, tongue, soft palate	7–26%
Lichen planus	Reticular, erosive, atrophic, bullous, ulcerative, popular, plaque like	Posterior buccal mucosa, tongue, gingivae, palate, vermilion border	0.4–3.7%
Discoid lupus erythematosus	White plaques with elevated borders, radiating white striae and telangiectasia	Cheeks, lips, palate	NA
Epidermolysis bullosa	Bullae and vesicle formation following mild trauma	Cheeks, tongue, palate	25%
Verruciform xanthoma	A well demarcated mass with a yellow-white or red color and a papillary or verruciform surface	Gingivae, tongue, buccal mucosa, vestibular mucosa, floor of the mouth	NA
Graft-versus host disease	Atrophy, erythema, erosions, ulcers, lichenoid lesions	Cheeks, tongue, lips, buccal & labial vestibule	NA

^*^NA: not assigned

### Leukoplakia (Leukokeratosis) 

Schwimmer^16^ first used this termin 1877 to describe a white plaque on the tongue. Since 1980, World Health Organization has changed the definition of leukoplakia as follows:^3^

1. A white patch or plaque that cannot be characterized clinically or histologically as any other disease.
2. A predominantly white lesion of the oral mucosa that cannot be characterized as any other definable lesion.
3. A white plaque of questionable risk having excluded (other) known disease or disorders that carry no increased risk for cancer.

This lesion is seen most often in middle-aged and older men. Less than 1% of males under 30 years of age have leukoplakia, but the prevalence increases to an alarming 8% in men and 2% in women over the age of 70.^10,17^ Although leukoplakia is more common in males than in females, the latter has a higher risk of developing oral cancer.^4^ Approximately 70% of oral leukoplakias are seen on the buccal mucosa, lip vermilion and gingivae. However, lesions on the floor of the mouth (42.9%), tongue (24.2%) and lip vermilion (24%) account for more than 90% of those with dysplastic or malignant changes. The rate of dysplastic or malignant alterations in oral leukoplakia has been reported to be between 15.6% and 39.2%. Leukoplakia usually occurs about 5 years earlier than OSCC.^4,10^Waldron and Shafer^18^demonstrated that 19.9% of leukoplakia had some degrees of dysplasia, 3.1% were frank carcinoma, 4.6% showed severe dysplasia or carcinoma in situ, and 12.2% showed mild to moderate dysplasia.

There are 4 subdivisions of leukoplakia: early or thin, homogenous or thick, granular or verruciform and speckled or erythroleukoplakia. Each subdivision has a different malignant transformation potential. For example, thin leukoplakia often becomes malignant without clinical changes. Thick leukoplakia undergoes malignant transformation in 1–7% of cases. The frequency of malignant changes in verruciform and speckled leukoplakia ranges from 4% to 15% and 18% to 47%, respectively.^4^

In a recent study, Liu et al^19^found that 20.8% of patients with verrucous leukoplakia developed cancer. They pointed out that age and lesion site were risk factors for malignancy, whereas sex, smoking, and alcohol intake were not. Leukoplakia exhibiting moderate to severe dysplasia warrants complete removal by electrocautery, cryosurgery or laser ablation. Leukoplakia not exhibiting dysplasia usually is not excised, but clinical assessment every 6 months is recommended.^4^

### Erythroplakia (Erythroplasia, Erythroplasia of Queyrat) 

Fournier and Darier^20^ first described erythroplasia as a malignant dyskeratosis with unknown etiology in 1893 and designated it as épithéliome papillaire. WHO defined oral erythroplakia or erythroplasia during the years as follows:^3^

1. Bright red, velvety plaques which cannot be characterized clinically or pathologically as being due to any other condition.
2. Red areas that cannot be diagnosed as any other definable lesion.
3. A predominantly red lesion of the oral mucosa that cannot be characterized as any other definable lesion.

Clinically, erythroplakia is classified into three types: homogeneous, granular, and speckled.^1^ Erythroplakia most frequently occurs in males aged 50–70. The point prevalence rate of erythroplakia in the oral cavity has been estimated to be 1 per 2500 adults. The lowest prevalence of erythroplakia was reported to be 0.01% by Lumerman et al^21^ and the highest was 0.2% according to Hashibe et al.^22^ Common sites of involvement are floor of the mouth, tongue, retromolar pad, and soft palate. This lesion is usually asymptomatic, but some patients may complain of a burning sensation.^4,10^

According to Shafer and Waldron,^23^ 51% of erythroplakias transformed into SCC, 40% were carcinoma in situ and 9% showed mild to moderate dysplasia. Because of 90% malignant transformation rate, early detection and immediate surgical excision are recommended. Early cancers of the oral cavity and lip (stage I, stage II) have a better prognosis; therefore, surgery or radiation are the treatments of choice. Chemotherapy is added to surgery and/or radiation in stage III and higher.^4^

### Proliferative Verrucous Leukoplakia 

Proliferative verrucous leukoplakia (PVL) is a distinct and aggressive form of oral leukoplakia, which was first described by Hansen et al^24^ in 1985. Most patients with PVL are women (female-to-male ratio of 4:1) without any history of tobacco use. Clinically, PVL presents on the oral mucosa as a multifocal white patch or plaque with rough surface projections. These lesions tend to spread slowly and involve other oral mucosal regions. PVL occurs more frequently on the gingiva; however, other sites may be affected as well.^4^Bagan et al^25^ and Batsakis et al^26^ found SCC development in 63.3% and 100% of PVL, respectively. Transformation into SCC usually occurs within 8 years of initial PVL diagnosis.^4^ PVL is also characterized by resistance to generally approved treatments such as surgery, CO_2_ laser evaporation, laser surgery, chemotherapy and radiotherapy.^27^

###  Viadent Leukoplakia (Sanguinaria-associated Keratosis)

Viadent leukoplakia is a white patch or plaque, which is associated to sanguinaria mouth rinse. Sanguinaria extract is a mixture of benzophenanthridine alkaloids derived from bloodroot plant.^28^ According to Damm et al^29^ an increased prevalence of leukoplakia has been observed in the maxillary alveolar mucosa in patients using sanguinaria mouthwash. It seems that sanguinaria extract has a carcinogenic effect. Therefore, viadent leukoplakia should be considered a premalignant lesion and mouth rinses containing sanguinaria should be avoided until the risk for transformation into malignancy is determined.^30^

### Candida Leukoplakia (Candida Hyperplasia, Chronic Hyperplastic Candidiasis) 

*Candida *leukoplakia (CL) is a chronic form of candidiasis which is characterized by firm, white, leathery plaques on the cheeks, lips, palate and tongue. Generally, epithelial dysplasia occurs four to five times more frequently in CL than in leukoplakia.^28^

### Smokeless Tobacco Keratosis (Snuff Dipper’s Lesion, Spit Tobacco Keratosis, Tobacco Pouch Keratosis)

Smokeless tobacco keratosis is characterized by a white plaque in the buccal or labial vestibule where the tobacco is held. The degree of mucosal alteration depends on the quality and type of tobacco, the frequency of tobacco use and host susceptibility. This entity has been observed in 60% of snuff dippers and 15% of chewing tobacco users. Chewing tobacco or snuff is usually started at 8–14 years of age. Smokeless tobacco keratosis takes about 5 years to develop. Epithelial dysplasia has been found in this lesion and the risk of developing OSCC is four times greater in smokeless tobacco users compared to non-users. In 98% of patients, breaking the habit leads to a normal mucosa within 2 to 6 weeks. A lesion that remains more than 6 weeks should be considered a true leukoplakia or carcinoma and should be biopsied. ^4,31-33^

### Palatal Keratosis associated with Reverse Smoking

Reverse smoking is seen in patients of low socioeconomic class and is more common in females (91.3%), especially after the third decade of life. The frequency of oral involvement is high (97.8%), and the most commonly affected areas are the palate and the tongue.^34^ According to Baric et al^35^the risk of malignant transformation in oral lesions of reverse smokers was 19 times more than that of nonsmokers. In addition, Alvarez-Gómez et al^34^ found epithelial dysplasia and OSCC in 83.3% and 12.5% of reveres smokers, respectively.

### Verrucous Hyperplasia

Verrucous hyperplasia (VH) was first described as an extensive, thick, white plaque by Shear and Pindborg^36^in 1980. There are histopathological differences between VH and verrucous carcinoma. VH is characterized by the thickened epithelium with superficial to adjacent normal epithelium, whereas verrucous carcinoma is characterized by an invaded pushing border of the hyperplastic epithelium to connective tissue, but the basement membrane is intact. In addition, Zhu et al^37^ demonstrated that VH located on the lower lip had a higher risk of malignant transformation compared with other sites. Despite the rarity of VH in Western countries, it is a quite common oral premalignant lesion in Taiwan. The approved treatment is surgical excision. However, photodynamic therapy has also been recommended.^38^

### Oral Verrucous Carcinoma (Snuff Dipper’s Cancer, Ackerman’s Tumor)

Oral verrucous carcinoma (OVC) is a low-grade variant of OSSC, which was first described in 1984. It has been demonstrated that 1–10% of all OSCCs are OVC. The incidence rate of OVC has been estimated as one oral lesion per 1,000,000 of the population each year. OVC predominantly occurs in patients with the habit of areca chewing, cigarette smoking and alcohol drinking. Malignant transformation has been found in 20% of OVC cases. Surgical excision, radiotherapy and chemotherapy have been recommended for treatment of OVC.^4,38^

### Dyskeratosis Congenita (Cole-Engman Syndrome or Zinsser-Cole-Engman Syndrome)

Dyskeratosis congenita (DC) is a rare inherited disease, which is characterized by the classic triad of nail dystrophy, reticular skin pigmentation, and oral leukoplakia.^39^DC may affect one in 1,000,000 of the population.^40^ It mainly occurs in men (male-to-female ratio of 13:1) and manifests between 5 and 13 years of age.^39^Oral lesions, as white keratotic patches, occur in about 80% of cases. Typically, the buccal mucosa, tongue and oropharynx are affected. Ectodermal anomalies and neutropenia may lead to severe periodontal destruction. DC has an increased potential of malignant transformation.^39-41^ In a recent case report, Ray et al^41^found malignant transformation on the patients’ tongue with DC. There is no effective treatment for DC. However, surgery and treatment with bleomycin, vitamin A, steroids and testosterone have been recommended for the management of this condition.^39^

### Actinic Cheilosis (Actinic Cheilitis)

Actinic cheilosis (AC) is a premalignant condition frequently involving the lower lip. The main clinical presentations of AC are diffuse poorly-defined atrophic, erosive, ulcerative or keratotic plaques. Other clinical signs are dryness, scaly patches, swelling, transverse fissures, crusting and blotching.^42,43^ There is a strong likelihood of progressing to a malignancy. AC is one of the main causes of lip cancer, which is regarded as the 10 most common cancers in men. Six to ten percent of AC cases evolve into SCC over time.^4,43,44^

In a recent study, Kwon et al^42^ demonstrated that lip SCC originating from AC is more prone to metastasis than SCC arising from its cutaneous counterpart. Furthermore, they pointed out that the presence of bleeding, indurations, disease recurrence and persistent pain should be considered as markers of AC transformation into SCC.

There are various treatment methods for AC, including surgery, cryotherapy, electrosurgery, topical retinoids, 5-flurouracil cream, photodynamic therapy, CO_2_ laser ablation and vermilionectomy.^4,43,44^

### Keratoacanthoma (Self-healing Carcinoma, Pseudocarcinoma, Keratocarcinoma)

Keratoacanthoma (KA) was first described in 1888. This entity appears as a firm, sessile, non-tender nodule with a central plug of keratin. It occurs more frequently on hair-bearing skin.^45^

The presence of this lesion was also reported on the lip and tongue by Ramos et al^46^ and Chen et al,^47^ respectively. According to Sanchez Yus et al^48^ at least 24% of KA showed malignant transformation into SCC. KA must be totally excised and studied for any evidence of malignancy.

### Oral Submucous Fibrosis

Oral submucous fibrosis (OSF) is a chronic, progressive, scarring, precancerous condition which is characterized by mucosal rigidity. It has been associated with long-lasting dipping of betel-nut or paan in the mouth. OSF predominantly occurs in Indians and Southeast Asians. This condition is more common in young adults, aged 20−40. The most frequently affected sites in the oral cavity are buccal mucosa, retromolar area, tongue and soft palate. Clinical signs and symptoms of OSF are vesicles, ulcerations, petechiae, melanoses, xerostomia, burning sensation, and limitation in jaw movement.^1,49^ Overall, patients with OSF are at least 19 times more likely to develop OSCC than healthy people.^4^

According to a recent study by Guo et al^50^ OSSC originating from OSF occurs at a younger age (45.8 vs. 55.9 years), is more common in men (male-to-female ratio 32.1:1 vs. 2.3:1) and is more invasive and potentially metastatic than OSSC not originating from OSF. Surgical excision is an accepted treatment method for OSF. Furthermore, intra-lesional injections of interferon-γ have been recommended.^4^

### Oral Lichen Planus

Lichen planus (LP) is a common chronic, immunologically mediated muco-cutaneous disease, which was first described in 1869. Most patients with lichen planus are middle-aged (over 40), and it is rare in children. Females account for at least 65% of patients. Cutaneous lichen planus is seen in 1% and oral lichen planus (OLP) affects 0.1 -2.2% of the population. Clinical variations of OLP are reticular, erosive, atrophic, bullous, ulcerative, papular and plaque-like. The most commonly affected site in the oral cavity is posterior buccal mucosa, followed by tongue, (lateral and dorsal), gingivae, palate and vermilion border. The risk of malignant change in OLP has been controversial for a long time and reported to be between 0.4% and 3.7%. Patients often experience this complication after 10 years.^4,51,52^ Hence, OLP should be followed for a long period. Topical or systemic corticosteroids are usually recommended for the management of OLP.^4,28^

### Discoid Lupus Erythematosus

Discoid lupus erythematosus (DLE) is a chronic, scarring, immunologic, mucocutaneous disease, which is characterized by white keratinized plaques with elevated borders, radiating white striae and telangiectasia.^53,54^ The prevalence of DLE is less than 5 in 10,000 individuals and is more common in women than men with female to male ratio of 1.8:1. The frequency of oral involvement in DLE is lower than skin lesions, and is found in about 20% of cases.^53^ Malignant transformation of DLE into SCC is usually observed on the lower lip, and in Caucasians.^55-57^ However, there are some case reports in African Americans.^54,55^ Ma et al^58^ demonstrated that 5.5% of lower lip SCCs arise from lesions of DLE. This rate was reported to be 6.8% by Liu et al.^53^ According to Liu et al^53^ epithelial dysplasia and age over 60 are risk factors for malignant transformation in DLE, whereas sex, location of the lesion, smoking and alcohol intake were not considered to be risk factors.^57^ Topical or systemic corticosteroids are usually recommended for management of DLE.^4^

### Epidermolysis Bullosa

Epidermolysis bullosa (EB) is a blistering disease of the skin and mucosa. Approximately 500,000 individuals are affected worldwide.^59^ There are three types of EB: simplex, junctional and dystrophic. Each type consists of several forms of disorders. Oral involvement has been reported in the junctional and dystrophic forms of the disease, which is characterized by bulla and vesicle formation following mild physical trauma.^60^ Malignant transformation of EB has been reported most commonly in junctional and dystrophic types. In the oral cavity, lingual mucosa is more susceptible to malignant transformation. Malignancy usually develops in areas of chronic ulceration during the second to third decades of life.^4^ According to Yuen and Jonkman,^61^ developing a SCC among adults with junctional EB is noticed in 25% of cases. Risk of SCC in EB has also been documented by Oliveira et al,^59^ Reichart,^60^ and Yuen and Jonkman.^61^ Moreover, Fine and Mellerio^62^ showed that EB is even at risk for developing basal cell carcinoma (BCC) and malignant melanoma. Topical or systemic corticosteroids are usually recommended for management of EB.

### Verruciform Xanthoma

Verruciform xanthoma (VX) is a rare benign lesion first described by Shafer^63^ in 1971. The lesions appear as a well-defined, asymptomatic, slightly elevated mass with a yellow-white or red color and a papillary or verruciform surface. It mainly occurs in the oral mucosa. The gingiva is the most commonly affected site, followed by tongue, buccal mucosa, vestibular mucosa, floor of the mouth, soft palate and lower lip. There is limited information about malignant transformation of VX. Mannes et al^64^ and Drummond et al^65^ described association of VX with SCC and carcinoma in situ, respectively.

Verruciform xanthoma is treated with conservative surgical excision. Recurrence after removal of the lesion is rare.^4^

### Graft-versus-host Disease

Graft-versus-host disease (GvHD) is a common complication in patients treated with allogenic peripheral stem cell transplantation.^66^ Oral involvement has been observed in 80% of patients, characterized by atrophy, erythema, erosion, ulcers, lichenoid lesions, xerostomia, and oral pain.^66,67^ Malignant transformations in oral lesions have been documented by Salum et al^68^ and Montebugnoli et al.^69^

Topical corticosteroids may facilitate the healing of focal oral ulcerations associated with GvHD. In some cases narcotic analgesics may be required.^4^

### Cheilitis Glandularis

Cheilitis glandularis (CG) is characterized by hyperplasia of minor salivary glands, first described by von Volkman^70^ in 1870. Frequently the lower lip is involved but lesions on the upper lip have also been reported. The main causes of CG are syphilis, bacterial infection, solar radiation, tobacco, poor oral hygiene and genetic factors.^71,72^ Transformation of CG into SCC has been found by Nico et al^73^ and Butt et al.^74^ Generally, a significant percentage of patients (18–36%) have been associated with the development of SCC of overlying epithelium of the lip.^75^

### Xeroderma Pigmentosum

Xeroderma pigmentosum (XP) is a rare autosomal recessive disease, which is associated with cutaneous malignant transformation. Oral involvement is usually observed in patients under 20.^4^ Squamous cell carcinoma of the lower lip and tongue has been reported in patients with XP by Chidzonga^76^ and Palattella.^77^Consequently, XP should be considered an oral premalignant disorder. Patients with XP are advised to avoid sunlight and unfiltered fluorescent light, and to wear appropriate protective clothing and sunscreens if they cannot avoid sun exposure.^4^

### Syphilis (Third Stage), Iron Deficiency Anemia (Plummer-Vinson or Patterson-Kelly Syndrome), Malnutrition, Vitamin A, B, and C Deficiency and Immunosuppressive Diseases (HIV)

Although the above conditions have been mentioned with malignant potential, data regarding their malignant transformation is controversial. However, they should be considered as predisposing factors of oral cancers.^78-80^

## Conclusion

Most cancers and their related complications are preventable through early detection. Unfortunately, oral premalignant disorders are usually misdiagnosed due to lack of adequate knowledge among the general population and even medical professionals. Therefore, improvement of physicians’ or dentists’ level of knowledge about oral PMDs may play a key role in saving patients’ lives.

## Acknowledgement

The authors would like to thank the staff members of Oral and Maxillofacial Department, the library, and the IT Department of Dental School, Shahid Beheshti University of Medical Sciences for their sincere cooperation.

## References

[1] George A,  BSS  BSS,  SS  SS, Varghese  SS, Thomas J, Gopakumar D, Mani V (2011). Potentially Malignant disorders of oral cavity. OMPJ.

[2] Little JW, Falace DA, Miller CS, Rhodus NL (2013). Dental management of the medically compromised patient. 8<sup>th</sup> ed.

[3] Amagasa T (2011). Oral premalignant lesions. Int Clin Oncol.

[4] Neville BW, Damm DD, Allen CR, Bouquot JE (2002). Oral and maxillofacial pathology. 2nd ed..

[5] Garsia M, Jemal A, Ward EM, Hao Y, Siegel RL, Thun MJ. Global cancer facts and figures 2007. Atlanta GA: American Cancer Society.

[6] Gloeckler Ries LA, Miller BA, Hankey BF, Kosary CL, Harras A, Edwards BK, eds. SEER cancer statistics review, 1973-1991. Bethesda, Md: US Department of Health and Human Services, Public Health Service, National Cancer Institue, 1994. Report no. NIH-94-2789.

[7] Day GL, Blot WJ (1992). Second primary tumors in patients with oral cancer. Cancer.

[8] Silverman S Jr, Gorsky M (1990). Epidemiologic and demographic update in oral cancer: California and national data--1973 to 1985. J Am Dent Assoc.

[9] Llewellyn CD, Johnson NW, Warnakulasuriya KA (2001). Risk factors for squamous cell carcinoma of the oral cavity in young people--a comprehensive literature review. Oral Oncol.

[10] Neville BW, Day TA (2002). Oral cancer and precancerous lesions. CA Cancer J Clin.

[11] Reibel J (2003). Prognosis of oral pre-malignant lesions: significance of clinical, histopathological, and molecular biological characteristics. Crit Rev Oral Biol Med.

[12] Epstein JB, Gorsky M, Cabay RJ, Day T, Gonsalves W (2008). Screening for and diagnosis of oral premalignant lesions and oropharyngeal squamous cell carcinoma: role of primary care physicians. Can Fam Physician.

[13] Andre K, Schraub S, Mercier M, Bontemps P (1995). Role of alcohol and tobacco in the aetiology of head and neck cancer: A case-control study in the Doubs region of France. Oral Oncol, Eur J Cancer.

[14] Warnakulasuriya S, Johnson NW, van der Waal (2007). Nomenclature and classification of potentially malignant disorders of the oral mucosa. J Oral Pathol Med.

[15] Paget J (1870). Cancer following ichthyosis of the tongue. Trans Clin Soc London.

[16] Schwimmer E (1877). Die idiopathischen Schleimhautplaques der Mundhöhle (Leukoplakia buccalis). Arch Dermat Syph.

[17] Bouquot JE, Gorlin RJ (1986). Leukoplakia, lichenplanus, and other oral keratoses in 23,616 white Americans over the age of 35 years. Oral SurgOral Med Oral Pathol.

[18] Waldron CA, Shafer WG (1975). Leukoplakia revisitedA clinicopathologic study 3256 oral leukoplakias. Cancer.

[19] Liu W, Shen XM, Liu Y, Li J, Zhou ZT, Wang LZ (2011). Malignant transformation of oral verrucous leukoplakia: a clinicopathologic study of 53 cases. J Oral Pathol Med.

[20] Fournier A, Darier, J. Épithéliome bénin syphilöide de la verge (épithéliome papillaire). Bull. Soc. franç. de dermat. et syph. 4:324,1893.

[21] Lumerman H, Freedman P, Kerpel S (1995). Oral epithelial dysplasia and the development of invasive squamous cell carcinoma. Oral Surg Oral Med Oral Pathol Oral Radiol Endod.

[22] Hashibe M, Mathew B, Kuruvilla B, Thomas G, Sankaranarayanan R, Parkin DM, Zhang ZF (2000). Chewing tobacco, alcohol, and the risk of erythroplakia. Cancer Epidemiol Biomarkers Prev.

[23] Shafer WG, Waldron CA (1975). Erythroplakia of the oral cavity. Cancer.

[24] Hansen LS, Olson JA, Silverman S Jr (1985). Proliferative verrucous leukoplakiaA long-term study of thirty patients. Oral Surg Oral Med Oral Pathol.

[25] Bagan JV, Jimenez Y, Sanchis JM, Poveda R, Milian MA, Murillo J, Scully C (2003). Proliferative verrucous leukoplakia: high incidence of gingival squamous cell carcinoma. J Oral Pathol Med.

[26] Batsakis JG, Suarez P, el-Naggar AK (1999). Proliferative verrucous leukoplakia and its related lesions. Oral Oncol.

[27] Gouvêa AF, Moreira AE, Reis RR, de Almeida OP, Lopes MA (2010). Proliferative verrucous leukoplakia, squamous cell carcinoma and axillary metastasis. Med Oral Patol Oral Cir Bucal.

[28] Greenberg MS, Glick MG (2002). Burket’s oral medicine: diagnosis and treatment. 10th ed.

[29] Damm DD, Curran A, White DK, Drummond JF (1999). Leukoplakia of the maxillary vestibule--an association with Viadent?. Oral Surg Oral Med Oral Pathol Oral Radiol Endod.

[30] Damm DD, Fantasia JE (2002). White patch of maxillary vestibuleSanguinarine-associated leukoplakia. Gen Dent.

[31] Madani AH, Dikshit M, Bhaduri D (2012). Risk for oral cancer associated to smoking, smokeless and oral dip products. Indian J Public Health.

[32] Underner M, Perriot J (2011). Smokeless tobacco. Rev Mal Respir.

[33] Gupta PC, Ray CS, Sinha DN, Singh PK (2011). Smokeless tobacco: a major public health problem in the SEA region: a review. Indian J Public Health.

[34] Alvarez Gómez GJ, Alvarez Martínez E, Jiménez Gómez R, Mosquera Silva Y, Gaviria Núñez AM, Garcés Agudelo A, Alonso Duque A, Zabala Castaño A, Echeverri González E, Isaac Millán M, Ramírez Ossa D (2008). Reverse smokers's and changes in oral mucosaDepartment of Sucre, Colombia. Med Oral Patol Oral Cir Bucal.

[35] Baric JM, Alman JE, Feldman RS, Chauncey HH (1982). Influence of cigarette, pipe, and cigar smoking, removable partial dentures, and age on oral leukoplakia. Oral Surg Oral Med Oral Pathol.

[36] Shear M, Pindborg JJ (1980). Verrucous hyperplasia of the oral mucosa. Cancer.

[37] Zhu LK, Ding YW, Liu W, Zhou YM, Shi LJ, Zhou ZT (2012). A clinicopathological study on verrucous hyperplasia and verrucous carcinoma of the oral mucosa. J Oral Pathol Med.

[38] Lin HP, Chen HM, Yu CH, Yang H, Wang YP, Chiang CP (2010). Topical photodynamic therapy is very effective for oral verrucous hyperplasia and oral erythroleukoplakia. J Oral Pathol Med.

[39] Auluck A (2007). Dyskeratosis congenitaReport of a case with literature review. Med Oral Patol Oral Cir Bucal.

[40] Scully C, Langdon J, Evans J (2012). Marathon of eponyms: 26 Zinsser-Engman-Cole syndrome (Dyskeratosis congenita). Oral Dis.

[41] Ray JG, Swain N, Ghosh R, Richa, Pattanayak Mohanty S. Dyskeratosis congenita with malignant transformation. BMJ Case Rep 2011;2011. DOI: 10.1136/bcr.03.2010.2848. 10.1136/bcr.03.2010.2848 PMC3029444 22715219

[42] Kwon NH, Kim SY, Kim GM (2011). A case of metastatic squamous cell carcinoma arising from actinic cheilitis. Ann Dermatol.

[43] Savage NW, McKay C, Faulkner C (2010). Actinic cheilitis in dental practice. Aust Dent J.

[44] Vieira RA, Minicucci EM, Marques ME, Marques SA (2012). Actinic cheilitis and squamous cell carcinoma of the lip: clinical, histopathological and immunogenetic aspects. An Bras Dermatol.

[45] Johnson HA (1968). Query: keratoacanthoma of mucous membrane? Plast Reconstr Surg 1968;41:373-5. Plast Reconstr Surg.

[46] Ramos LM, Cardoso SV, Loyola AM, Rocha MA, Durighetto-Júnior AF (2009). Keratoacanthoma of the inferior lip: review and report of case with spontaneous regression. J Appl Oral Sci.

[47] Chen YK, Lin LM, Lin CC, Chen CH (2003). Keratoacanthoma of the tongue: a diagnostic problem. Otolaryngol Head Neck Surg.

[48] Sánchez Yus E, Simón P, Requena L, Ambrojo P, de Eusebio E (2000). Solitary keratoacanthoma: a self-healing proliferation that frequently becomes malignant. Am J Dermatopathol.

[49] Angadi PV, Rekha KP (2011). Oral submucous fibrosis: a clinicopathologic review of 205 cases in Indians. Oral Maxillofac Surg.

[50] Guo F, Jian XC, Zhou SH, Li N, Hu YJ, Tang ZG (2011). A retrospective study of oral squamous cell carcinomas originated from oral submucous fibrosis. Zhonghua Kou Qiang Yi Xue Za Zhi.

[51] Abbate G, Foscolo AM, Gallotti M, Lancella A, Mingo F (2006). Neoplastic transformation of oral lichen: case report and review of the literature. Acta Otorhinolaryngol Ital.

[52] Cawson RA, Odell EW (2008). Cawson’s essentials of oral pathology and oral medicine. 8<sup>th</sup> ed.

[53] Liu W, Shen ZY, Wang LJ, Hu YH, Shen XM, Zhou ZT, Li J (2011). Malignant potential of oral and labial chronic discoid lupus erythematosus: a clinicopathological study of 87 cases. Histopathology.

[54] Alsanafi S, Werth VP (2011). Squamous cell carcinomas arising in discoid lupus erythematosus scars: unusual occurrence in an African-American and in a sun-protected area. J Clin Rheumatol.

[55] Harper JG, Pilcher MF, Szlam S, Lind DS (2010). Squamous cell carcinoma in an African American with discoid lupus erythematosus: a case report and review of the literature. South Med J.

[56] Voigtländer V, Boonen H (1990). Squamous cell carcinoma of the lower lip in discoid lupus erythematosus associated with hereditary deficiency of complement 2. Z Hautkr.

[57] Nair VL, Chacko M (1991). Disseminated discoid lupus erythematosus with squamous cell carcinoma. Indian J Dermatol Venereol Leprol.

[58] Ma D, Dai G, Guo C (1999). Carcinoma of the lips developing in discoid lupus erythematosus. Zhonghua Kou Qiang Yi Xue Za Zhi.

[59] Oliveira TM, Sakai VT, Candido LA, Silva SM, Machado MA (2008). Clinical management for epidermolysis bullosa dystrophica. J Appl Oral Sci.

[60] Reichart PA (2003). Oral precancerous conditions--an overview. Mund Kiefer Gesichtschir.

[61] Yuen WY, Jonkman MF (2011). Risk of squamous cell carcinoma in junctional epidermolysis bullosa, non-Herlitz type: report of 7 cases and a review of the literature. J Am Acad Dermatol.

[62] Fine JD, Mellerio JE (2009). Extracutaneous manifestations and complications of inherited epidermolysis bullosa: part IIOther organs. J Am Acad Dermatol.

[63] Shafer WG (1971). Verruciform xanthoma. Oral Surg Oral Med Oral Pathol.

[64] Mannes KD, Dekle CL, Requena L, Sangueza OP (1999). Verruciform xanthoma associated with squamous cell carcinoma. Am J Dermatopathol.

[65] Drummond JF, White DK, Damm DD, Cramer JR (1989). Verruciform xanthoma within carcinoma in situ. J Oral Maxillofac Surg.

[66] Demarosi F, Soligo D, Lodi G, Moneghini L, Sardella A, Carrassi A (2005). Squamous cell carcinoma of the oral cavity associated with graft versus host disease: report of a case and review of the literature. Oral Surg Oral Med Oral Pathol Oral Radiol Endod.

[67] Demarosi F, Lodi G, Carrassi A, Soligo D, Sardella A (2005). Oral malignancies following HSCT: graft versus host disease and other risk factors. Oral Oncol.

[68] Salum FG, Martins GB, de Figueiredo MA, Cherubini K, Yurgel LS, Torres-Pereira C (2006). Squamous cell carcinoma of the tongue after bone marrow transplantation in a patient with Fanconi anemia. Braz Dent J.

[69] Montebugnoli L, Gissi DB, Marchetti C, Foschini MP (2011). Multiple squamous cell carcinomas of the oral cavity in a young patient with graft-versus-host disease following allogenic bone marrow transplantation. Int J Oral Maxillofac Surg.

[70] von Volkman (1870). Einige falle von cheilitis glandularis apostematosa (Myxadenotis labialis). Arch Path Anat.

[71] Yanagawa T, Yamaguchi A, Harada H, Yamagata K, Ishibashi N, Noguchi M, Onizawa K, Bukawa H (2011). Cheilitis glandularis: two case reports of asian-Japanese men and literature review of Japanese cases. ISRN Dent.

[72] Taneja P, Singh N (2002). Cheilitis glandularis. Int Chin J Dent.

[73] Nico MM, Nakano de Melo, Lourenço SV (2010). Cheilitis glandularis: a clinicopathological study in 22 patients. J Am Acad Dermatol.

[74] Butt FM, Chindia ML, Rana FS, Ashani A (2007). Cheilitis glandularis progressing to squamous cell carcinoma in an hiv-infected patient: case report. East Afr Med J.

[75] Reiter S, Vered M, Yarom N, Goldsmith C, Gorsky M (2011). Cheilitis glandularis: clinico-histopathological diagnostic criteria. Oral Dis.

[76] Chidzonga MM, Mahomva L, Makunike-Mutasa R, Masanganise R (2009). Xeroderma pigmentosum: a retrospective case series in Zimbabwe. J Oral Maxillofac Surg.

[77] Palattella P (1970). Precancerous lesions of the face and mouth with particular reference to xeroderma pigmentosum. Ann Stomatol (Roma).

[78] Spornraft-Ragaller P, Boashie U, Friedrich K, Lehmann U, Meurer M (2006). Late secondary syphilis with ulceration of the tongue during HIV coinfection: case report. Hautarzt.

[79] Podlodowska J, Szumiło J, Podlodowski W, Starosławska E, Burdan F (2012). Epidemiology and risk factors of the oral carcinoma. Pol Merkur Lekarski.

[80] Peterlik M, Grant WB, Cross HS (2009). Calcium, vitamin D and cancer. Anticancer Res.

